# Creation of a Natural Interdigital Web in Toe Polysyndactyly With a Dorsal Trapezoidal and Plantar Triangular Flap Design

**DOI:** 10.7759/cureus.99746

**Published:** 2025-12-20

**Authors:** Daiki Ito, Ko Nakao

**Affiliations:** 1 Plastic Surgery, Subaru Health Insurance Society Ota Memorial Hospital, Ota, JPN; 2 Plastic and Reconstructive Surgery, Subaru Health Insurance Society Ota Memorial Hospital, Ota, JPN

**Keywords:** dorsal trapezoidal flap, interdigital web reconstruction, polysyndactyly, skin graft, toe surgery

## Abstract

Surgical correction of polysyndactyly of the toes requires not only functional separation of the digits but also creation of an aesthetically acceptable interdigital web, as postoperative appearance strongly affects psychosocial outcomes. Although traditional techniques, including Z-plasty and dorsal rectangular flaps as well as web-preserving flaps with or without skin grafting, have been described, complications such as web creep, wound dehiscence, and valgus deformity of the residual toes remain problematic.

Here, we report a case of polysyndactyly involving the fourth, fifth, and sixth toes of the left foot in a five-year-old girl. A trapezoidal dorsal rectangular flap was created in the fourth interdigital space and combined with a triangular plantar flap to achieve a web that was narrow in the mid-portion and broader at the base. The dorsal flap base and plantar inset were advanced 2 mm proximally to enhance web depth. Residual skin defects were covered using a full-thickness skin graft harvested from the medial malleolar region. Six months postoperatively, the reconstructed web showed appropriate depth, tapering, and alignment consistent with the adjacent interdigital spaces. No valgus deviation was observed in the residual toes. The skin graft demonstrated excellent take with only minor pigmentation differences and no significant scar contracture or web creep. The patient’s postoperative function and ambulation were uneventful.

The combination of a trapezoidal dorsal rectangular flap and plantar triangular flap with proximal advancement of the inset facilitates the creation of a deep and natural-appearing interdigital web. Adjunctive full-thickness skin grafting allows tension-free closure of larger skin deficits, reducing the risk of secondary complications. Although this report describes a single case, the technique may be useful in the aesthetic reconstruction of polysyndactyly of the toes.

## Introduction

In the surgical management of syndactyly and polysyndactyly of the toes, functional reconstruction as well as aesthetic outcomes are of paramount importance, as they considerably affect patient psychological health and quality of life. This is particularly relevant in cultures where barefoot ambulation is common because deformities or an unattractive appearance of the toes may result in considerable social and psychological distress [1].

Traditional techniques such as Z-plasty and dorsal rectangular flaps have been described for web space reconstruction [2]. More recently, alternative approaches, including interdigital web-preserving flaps and the adjunctive use of skin grafts, have been adopted [3]. Nevertheless, postoperative complications such as web creep and valgus deformity of the residual toe remain critical aesthetic issues [4,5].

We present a novel surgical technique that combines trapezoidal dorsal and triangular plantar flaps. This design allows the creation of an interdigital web that is narrow at its mid-portion and wider at its base, thereby reproducing a more natural toe contour. In addition, a deeper and more stable web could be achieved by positioning the flap inset line more proximally. We describe the design, operative techniques, and postoperative outcomes of this approach.

## Case presentation

**Figure 1:**
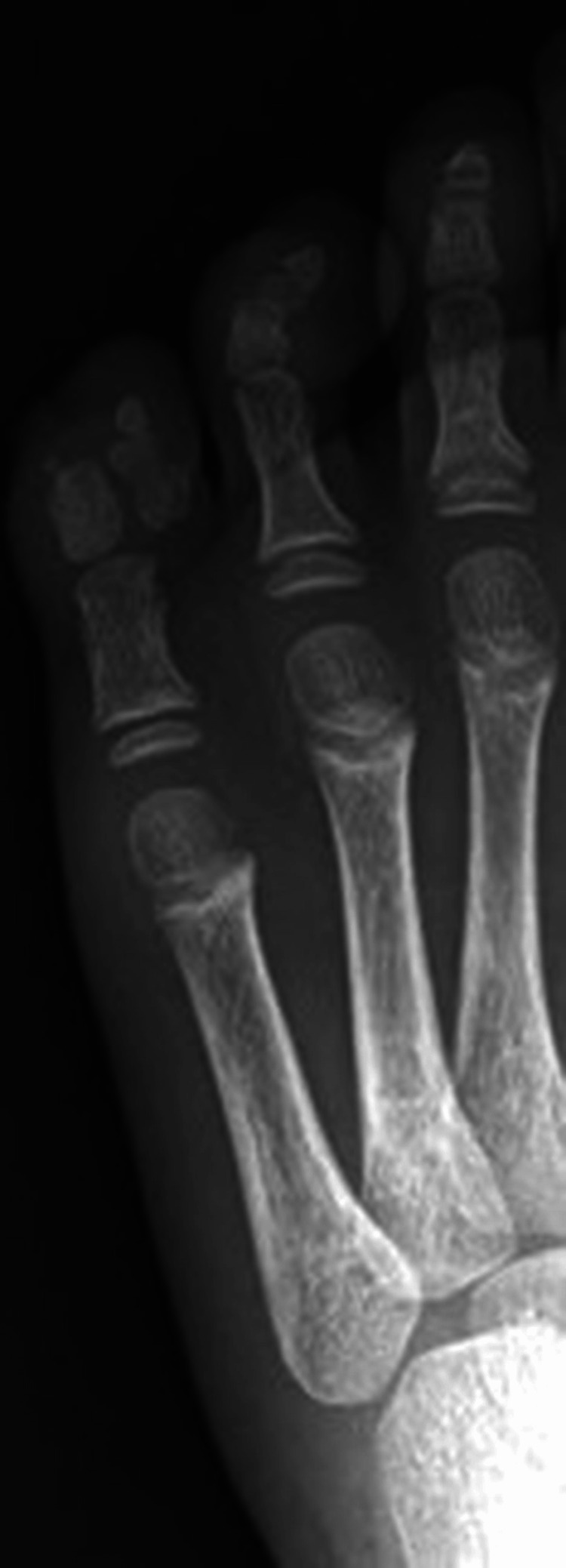
Preoperative radiograph of the left foot demonstrating polysyndactyly of the third, fourth, and fifth toes (right to left) Observed on the left foot is polysyndactyly of the third, fourth, and fifth (right to left) toes with medial deviation and duplication of the fifth toe (extreme left), which was selected for excision.

A five-year-old girl had polysyndactyly involving the third, fourth, and fifth toes of the left foot from birth. She was initially evaluated at three years of age, but definitive surgery was postponed until the age of five to ensure postoperative compliance with cast immobilization and rest. Radiography at four years demonstrated a medial deviation of the fifth toe relative to the longitudinal axis, along with duplication of the lateral rays (Figure 1). Based on these findings, the fifth toe was judged to be redundant and was selected for excision.

Surgical design

**Figure 2:**
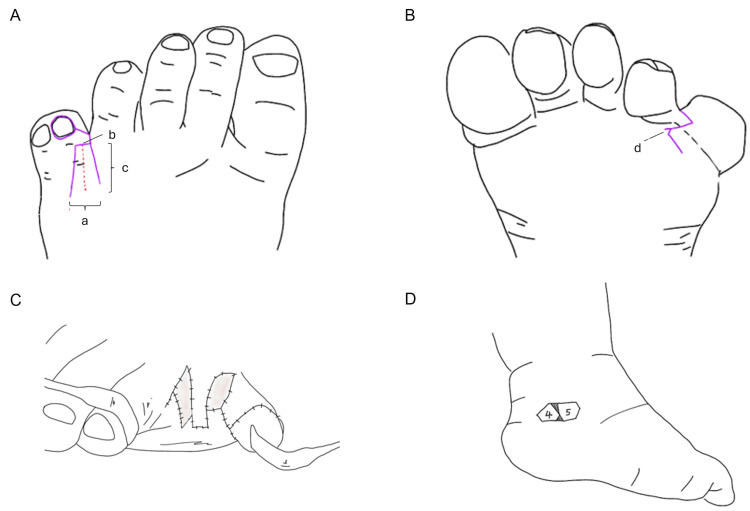
Schematic illustration of the flap design and graft placement A: Dorsal view of the fourth web space demonstrating the trapezoidal dorsal flap. Key measurements indicated are (a) base width of the dorsal flap, (b) distal width of the dorsal flap, and (c) flap length. B: Plantar view showing the zigzag incision line (d) used for plantar flap elevation; C:  Intraoperative view after flap elevation and defect preparation; D: Full-thickness skin graft harvested from the ipsilateral medial malleolar region and applied to areas not covered by local flaps

A trapezoidal dorsal flap measuring 16 mm in length (Figure 2A, c), with a base width of 10 mm (Figure 2A, a) and a distal width of 8 mm (Figure 2A, b), was created in the fourth interdigital web space (Figure 2A). A straight incision was made along the dorsal web, and a zigzag plantar incision was designed to facilitate elevation of the plantar triangular flap (Figure 2B, d). Skin from the plantar surface of the excised fifth toe was deemed suitable for coverage of the lateral defect of the fourth toe.

**Figure 3:**
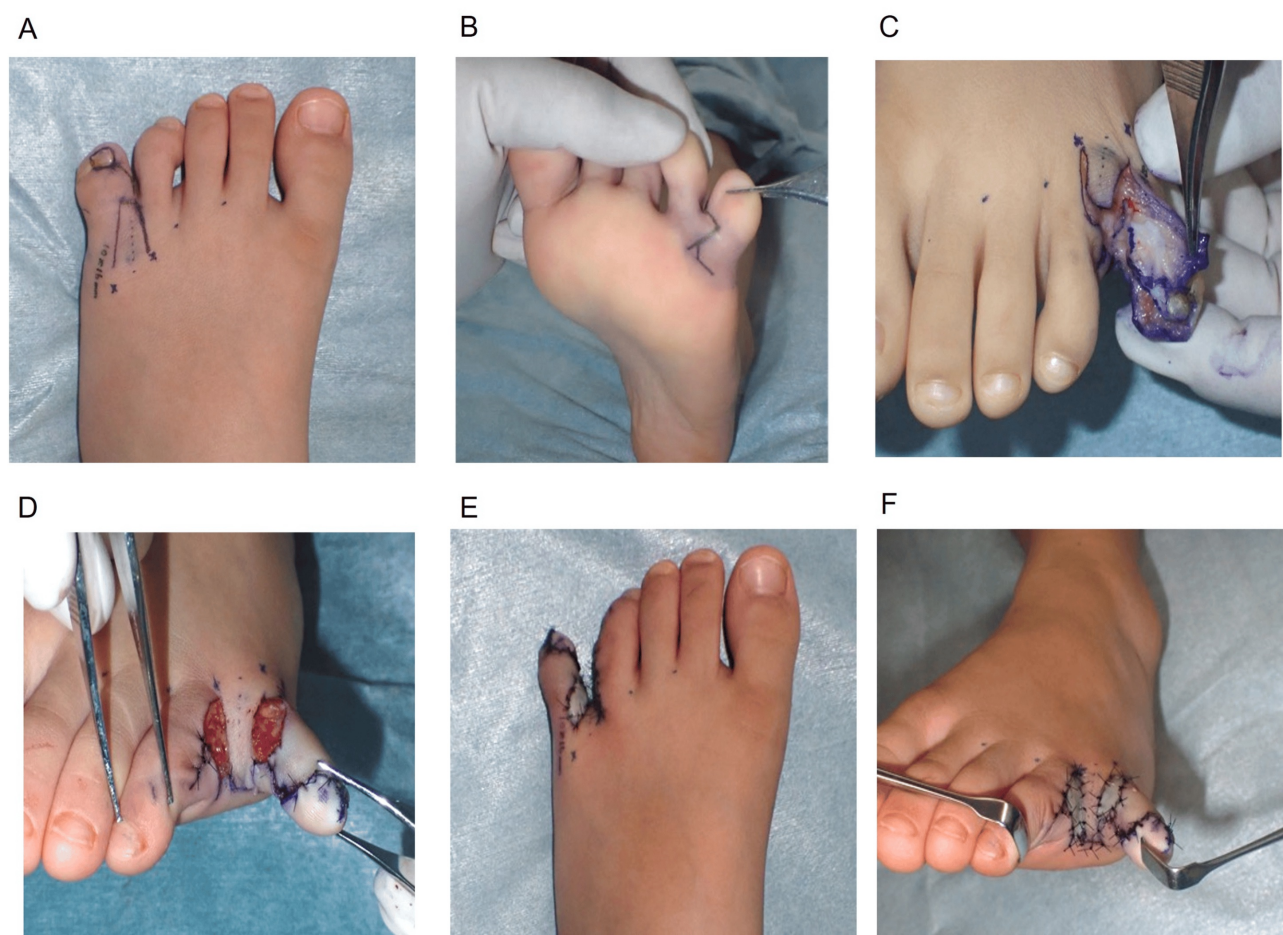
Schematic design of the dorsal and plantar flaps for reconstruction of the fourth interdigital web A: Dorsal view showing the planned trapezoidal dorsal flap design; B: Plantar view illustrating the zigzag incision line used for plantar flap elevation; C: Orientation of the flap components before inset; D: Final configuration of the dorsal and plantar flaps following inset; E: Geometric characteristics of the trapezoidal dorsal flap and triangular plantar flap after flap inset, demonstrating the final dimensional relationships of the reconstructed web; F: Illustration demonstrating how the combined flap design achieves a narrow mid-portion and a widened web base, reproducing a natural interdigital contour

To achieve a deep interdigital web, both the dorsal flap base and the plantar flap inset line were positioned 2 mm proximally relative to the intended web line (Figure 3A). The dorsal flap was shaped as a trapezoid to narrow the central portion of the web, while a 2 mm triangular plantar flap (Figure 3B) was incorporated to widen the web base and maintain a natural tapering contour (Figures 3C-3F). Areas of skin deficiency that could not be closed with local flaps were reconstructed using a full-thickness skin graft harvested from the ipsilateral medial malleolar region (Figures 2C-2D).

The medial malleolus was selected as the donor site because its skin has high pliability and is well-suited for harvesting a graft of appropriate quality and thickness. To prevent contour deformities such as depression or bulging at the harvest site, as well as graft necrosis associated with differences in vascularity between the donor and recipient areas, a bulky pressure dressing and splint immobilization were applied postoperatively for two weeks. In addition, tape therapy was initiated after immobilization to promote scar maturation and to maintain the contour of both the donor site and the reconstructed web.

Postoperative care protocol

Postoperative immobilization was achieved using a short-leg cast, and the patient remained hospitalized until postoperative day five to ensure strict rest and appropriate monitoring. On postoperative day 13, the cast was removed under general anesthesia, and sutures were removed at the same time. Immobilization was then converted to a cast splint to maintain the reconstructed web space while allowing gradual soft-tissue adaptation. The splint was discontinued on postoperative day 25, after which full weight-bearing on the affected limb was permitted. Thereafter, tape therapy was initiated to support scar maturation, minimize hypertrophic scarring, and preserve the depth and contour of the reconstructed interdigital web.

Postoperative course

**Figure 4:**
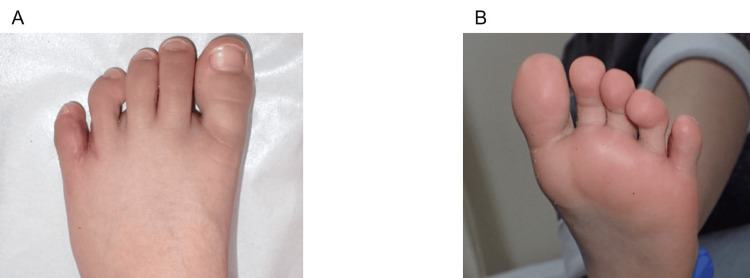
Postoperative clinical outcome six months after reconstruction of the fourth interdigital web A: Dorsal view showing a deep and well-formed web with normal toe alignment; B: Plantar view demonstrating stable reconstruction, good graft take, and no scar contracture or pigmentation abnormalities

Six months postoperatively, the reconstructed web depth and toe alignment were comparable to those of the adjacent normal interdigital spaces. No valgus deviation was observed in the residual toes. The skin graft results were satisfactory, with no conspicuous scar contracture or pigmentation abnormalities. The patient’s postoperative function and ambulation were unremarkable (Figures 4A-4B).

## Discussion

The goals for surgical correction of polysyndactyly include not only functional separation of the digits but also the creation of a deep and aesthetically acceptable interdigital web. The Z-plasty has historically been the most commonly used technique [2]. Based on this concept, modifications using dorsal triangular or rectangular flaps and interdigital web-preserving flaps have been described. However, conventional rectangular flaps often leave redundant bulk at the central web, resulting in an unnaturally widened interdigital space and predisposition to valgus deformity of the residual toe.

A study has shown that postoperative web creep and valgus deviation may occur following conventional reconstruction techniques [4]. Although these approaches can stabilize the metatarsophalangeal joint, they do not address the broad appearance of the web. In the present case, the creation of a trapezoidal dorsal flap reduced the central bulk, yielding a narrower and more natural web shape, while minimizing valgus deformity. The addition of a plantar triangular flap allowed for a more fan-shaped, widened web base. A deeper web was established by positioning the flap inset 2 mm proximally to the intended web line.

Full-thickness skin grafting was performed to cover residual skin defects and to avoid excessive tension or forced primary closure, which could otherwise lead to wound dehiscence or contracture. Although graftless techniques, including the dorsal pentagonal island flap [6] and double volar flap method [7], have been described, they risk flap congestion due to overstretching and are associated with complications, such as web creep, flexion contracture, and wound dehiscence [8]. From the long-term perspective, the adjunctive use of full-thickness skin grafts continues to offer good stability.

With respect to postoperative scarring, O'Connor et al. reported that when appropriate taping and ultraviolet protection were implemented, donor-site scars and trapdoor deformities of the grafted sites were minimal [9]. As observed in these studies, the careful postoperative management of our patient also resulted in inconspicuous graft scarring and pigmentation. The current report describes a single case, and further clinical experience and long-term follow-up are required to validate the effectiveness and stability of this novel technique.

## Conclusions

The combination of a dorsal trapezoidal and plantar triangular flap with proximal advancement provides a straightforward and reliable method for reconstructing a deep, natural-appearing interdigital web in toe polysyndactyly. This technique allows tension-free closure, minimizes complications such as web creep and valgus deformity, and achieves excellent aesthetic and functional outcomes. Although demonstrated in a single case, it represents a practical and reproducible option for surgeons seeking both functional and cosmetic refinement in complex toe reconstruction.
